# Supplementary material for: miRNA-mediated control of gephyrin synthesis drives sustained inhibitory synaptic plasticity
Source: EMBO Rep. 2024 Sep 18;25(11):5141–68. doi: 10.1038/s44319-024-00253-z (PMC11549329; doi:10.1038/s44319-024-00253-z)
Supplement: Supplementary file 2 — Expanded View Figures [file 44319_2024_253_MOESM2_ESM.pdf]

Expanded View Figures

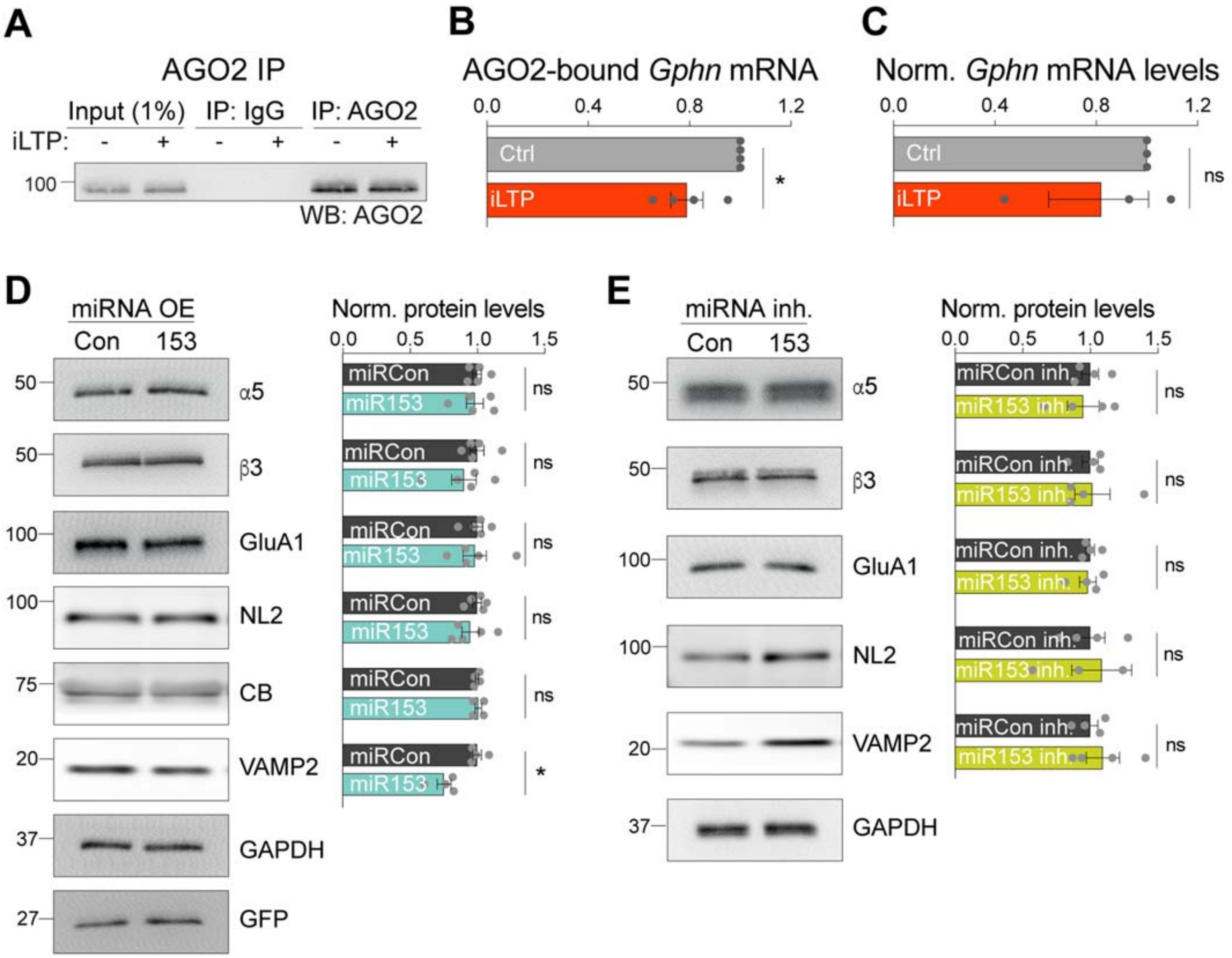

Figure EV1. Control experiments for AGO2 IPs and impact of miR153 on other synaptic proteins.

(**A**) Western blot (WB) of AGO2 immunoprecipitated from neurons following control treatment (Ctrl) or 90 min post-iLTP stimulation (iLTP). (**B**) qRT-PCR of *Gphn* mRNA bound to AGO2 in neurons from (**A**). AGO2-bound *Gphn* was normalized to total *Gphn* mRNA expression, and fold change from Ctrl was quantified for each condition. $N = 4$. $P = 0.0452$. (**C**) qRT-PCR of total *Gphn* mRNA levels in Ctrl and iLTP-90 neurons. *Gphn* mRNA levels were normalized to U6 expression, and fold change from Ctrl was quantified for each condition. $N = 3$. $P = 0.7500$. (**D**) Left: western blots of $GABA_AR$ subunits α5 (extrasynaptic) and β3 (synaptic), AMPAR subunit GluA1, GPHN binding proteins neuroligin-2 (NL2) and collybistin (CB), miR153 target VAMP2, GAPDH, and GFP protein levels in neurons overexpressing miRCon or miR153miRNA overexpression (OE) constructs contain a GFP reporter. Right: quantification of α5, β3, GluA1, NL2, CB, VAMP2 in miRCon or miR153 OE neurons. Protein levels were normalized to GAPDH, and the data quantified as relative change in normalized protein expression. $N = 5$. *P*-values (miRCon vs miR153): α5 > 0.9999, β3 = 0.5476, GluA1 = 0.5476, NL2 = 0.4206, CB > 0.9999, VAMP2 = 0.0286. (**E**) Left: western blots of α5, β3, GluA1, NL2, VAMP2, and GAPDH protein levels in neurons expressing miRCon or miR153 inhibitors. Right: quantification of α5, β3, GluA1, NL2, CB, VAMP2 in miRCon neurons or neurons in which miR153 was inhibited. Protein levels were normalized to GAPDH, and the data quantified as relative change in normalized protein expression. $N = 4$. *P*-values (anti-Con vs anti-153): α5 = 0.8857, β3 = 0.8857, GluA1 = 0.8857, NL2 = 0.8857, CB > 0.9999, VAMP2 = 0.6857. *N* = independent neuronal cultures/experiments. All values represent mean ± SEM. *$p < 0.05$, **$p < 0.01$, ***$p < 0.005$, ****$p < 0.0001$; one-sample t-test (**B**), Wilcoxon signed rank test (**C**), and Mann–Whitney test (**D**, **E**).

 © The Author(s)

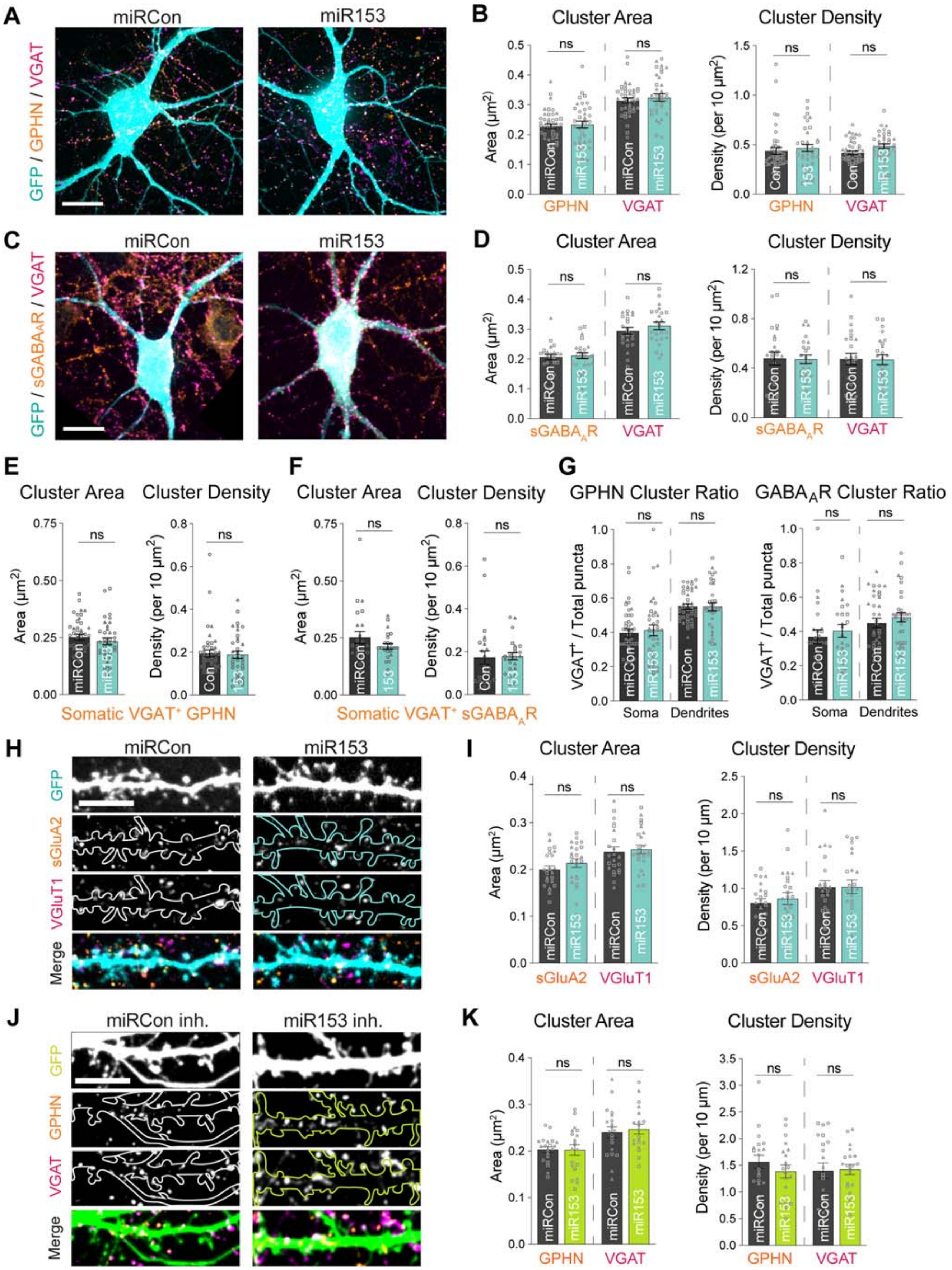
A
miRCon
miR153
GFP / GPHN / VGAT
B
Cluster Area
Cluster Density
ns
Area (μm²)
Density (per 10 μm²)
GPHN
VGAT
C
GFP / sGABAAR / VGAT
D
sGABAAR
E
F
G
Somatic VGAT⁺ GPHN
Somatic VGAT⁺ sGABAAR
GPHN Cluster Ratio
GABAAR Cluster Ratio
VGAT⁺ / Total puncta
Soma
Dendrites
H
GFP
sGluA2
VGluT1
Merge
I
Density (per 10 μm)
J
miRCon inh.
miR153 inh.
K

Figure EV2. Control experiments for impact of miR153 manipulations on inhibitory somatic synapses and glutamatergic synapses.

(**A**) Representative somata of miRCon or miR153 OE-expressing neurons labeled with antibodies to GPHN and VGAT. Scale bar, 80 µm. (**B**) Quantification of GPHN and VGAT cluster area (left) and cluster density (right) in neurons from (**A**). $N = 3$ / $n = 37–43$ neurons per condition. *P*-values (miRCon vs miR153): GPHN area = 0.7738, VGAT area = 0.6588, GPHN density = 0.7784, VGAT density = 0.3919. (**C**) Representative somata of miRCon or miR153 OE-expressing neurons labeled with antibodies to surface $GABA_AR$ γ2 subunit ($sGABA_AR$) and VGAT. Scale bar, 80 µm. (**D**) Quantification of $sGABA_AR$ and VGAT cluster area (left) and cluster density (right) in neurons as shown in (**C**). $N = 3$ / $n = 32–35$ neurons in each condition. *P*-values (miRCon vs miR153): γ2 area = 0.7593, VGAT area = 0.4208, γ2 density = 0.9159, VGAT density = 0.9221. (**E**) Quantification of $VGAT^+$ GPHN cluster area (left) and density (right) from total GPHN puncta quantified in (**B**). $N = 3$ / $n = 37–43$ neurons per condition. *P*-values (miRCon vs miR153): GPHN area = 0.3569, GPHN density = 0. 8065. (**F**) Quantification of $VGAT^+$ $sGABA_AR$ cluster area (left) and density (right) from total $sGABA_AR$ puncta quantified in (**D**). $N = 3$ / $n = 32-35$ neurons per condition. *P*-values (miRCon vs miR153): γ2 area = 0.3028, γ2 density = 0.8555. (**G**) Proportion of $VGAT^+$ GPHN clusters (left) and $VGAT^+$ $sGABA_AR$ clusters (right) in soma and dendrites. $N = 3$ / $n = 32–42$ neurons. *P*-values (miRCon vs miR153): somatic GPHN = 0.7279, dendritic GPHN = 0.9519, somatic γ2 = 0.5695, dendritic γ2 = 0.3768 (**H**) Representative dendritic segments of miRCon or miR153 OE neurons labeled with antibodies to surface AMPAR subunit GluA2 (sGluA2) and VGluT1. Scale bar, 10 µm. (**I**) Quantification of surface GluA2 and VGluT1 cluster area (left) and cluster density (right) in neurons from (**H**). $N = 3$ / $n = 23–24$ neurons per condition. *P*-values (miRCon vs miR153): sGluA2 area = 0.2599, VGluT1 area = 0.7703, sGluA2 density = 0.5529, vGluT1 density = 0.9867. (**J**) Representative dendritic segments of miRCon or miR153 inhibitor-expressing neurons labeled with antibodies to gephyrin (GPHN) and VGAT. Scale bar, 10 µm. (**K**) Quantification of GPHN and VGAT cluster area (left) and cluster density (right) in neurons from (**A**). $N = 4$ / $n = 20$ neurons per condition. *P*-values (anti-Con vs anti-153): GPHN area = 0.9630, VGAT area = 0.7950, GPHN density = 0.5259, VGAT density = 0.9461. *N* = independent neuronal cultures/experiments, *n* = neurons. All values represent mean ± SEM. Neurons from different culture preparations are represented by different symbols of data points. $*p < 0.05$, $**p < 0.01$, $***p < 0.005$, $****p < 0.0001$; nested t-test.

 © The Author(s)

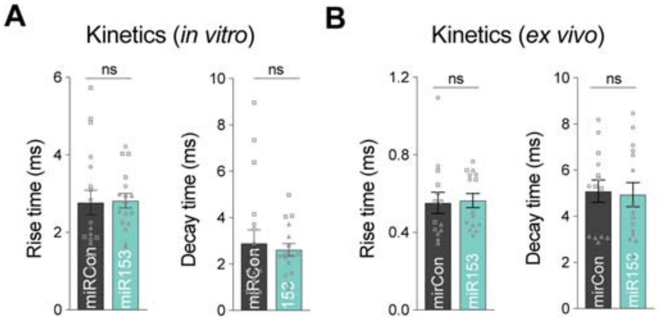

Figure EV3. Measurement of mIPSC kinetics.

(**A**) Quantification of mIPSC rise time (left) and decay time (right) from miRCon and miR153 OE-expressing neurons in culture. $N = 3$ / $n = 17–18$ neurons per condition. *P*-values (miRCon vs miR153): rise time = 0.7836, decay time = 0.9860 (**B**) Quantification of mIPSC rise time (left) and decay time (right) from miRCon and miR153 OE-expressing neurons in slice. $N = 3–4$ / $n = 17–22$ neurons per condition. *P*-values (miRCon vs miR153): rise time = 0.6531, decay time = 0.8593. N = independent neuronal cultures/experiments, n = neurons. All values represent mean ± SEM. Neurons from different neuronal preparations are represented by different symbols of data points. $*p < 0.05$, $**p < 0.01$, $***p < 0.005$, $****p < 0.0001$; nested t-test (**A**) and Mann–Whitney test (**B**).

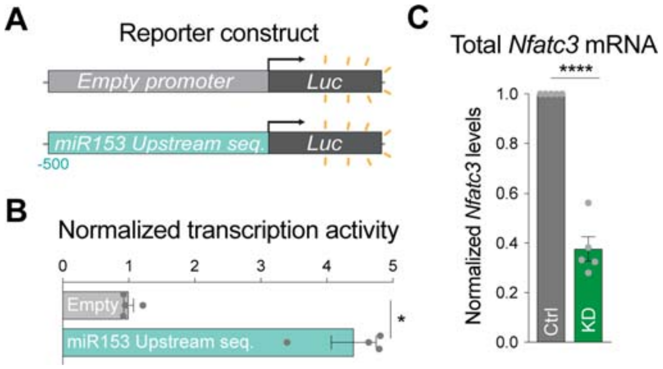

Figure EV4. Control experiments for Luc reporter construct and NFATc3 knockdown construct.

(**A**) Schematic of the Empty-Luc (no promoter) and miR153^{-500}-Luc luciferase reporters, designed to test transcriptional activity of the sequence 500 bp upstream of miR153. (**B**) Quantification of Luc activities in neurons expressing reporters containing no promoter (Empty) or the sequence upstream of pri-miR153 coding region (miR153 Upstream seq.). Firefly was normalized to Renilla, and the data quantified as relative change in normalized Luc activity with error-corrected control values. $N = 4$. $P = 0.0286$. (**C**) qRT-PCR of total *Nfatc3* mRNA levels in Ctrl and NFATc3 knockdown (NFAT KD) neurons. *Nfatc3* mRNA levels normalized to *Gapdh* mRNA expression, and fold change from Ctrl was quantified for each condition. $N = 6$. $P = 0.0002$. N = independent neuronal cultures/experiments. All values represent mean ± SEM. *$p < 0.05$, **$p < 0.01$, ***$p < 0.005$, ****$p < 0.0001$; Mann–Whitney test (**B**) and one-sample t-test (**C**).

 © The Author(s)

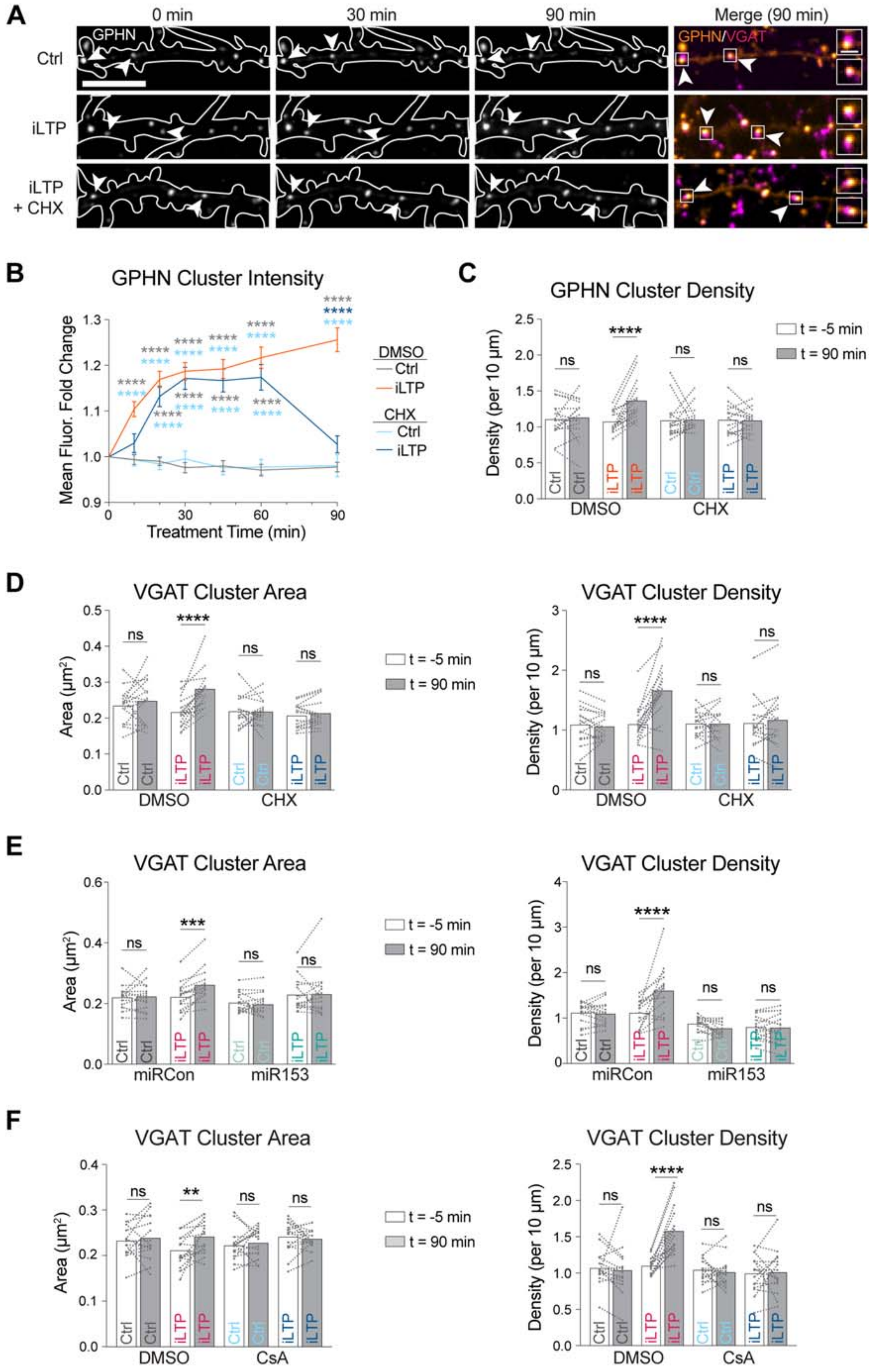
A
0 min
30 min
90 min
Merge (90 min)
GPHN
GPHN/VGAT
Ctrl
iLTP
iLTP + CHX
B
GPHN Cluster Intensity
Mean Fluor. Fold Change
Treatment Time (min)
DMSO
Ctrl
iLTP
CHX
Ctrl
iLTP
C
GPHN Cluster Density
Density (per 10 µm)
t = -5 min
t = 90 min
DMSO
CHX
D
VGAT Cluster Area
Area (µm²)
VGAT Cluster Density
Density (per 10 µm)
DMSO
CHX
E
VGAT Cluster Area
VGAT Cluster Density
miRCon
miR153
F
VGAT Cluster Area
VGAT Cluster Density
DMSO
CsA

Figure EV5. Control experiments for inhibitory synapse live imaging.

(**A**) Representative dendritic segments of neurons expressing GPHN IB and labeled with an antibody to VGAT, imaged over time in control and iLTP conditions in the presence or absence of translational inhibitor cycloheximide (CHX). Puncta are labeled with filled arrowheads when the fluorescence is unchanged and open arrowheads when fluorescence increases over time. Boxes indicate the fluorescent puncta enlarged in the merged images (dendrite scale bar, 10 μm; synapse scale bar, 2 μm). (**B**) Quantification of fold change in GPHN puncta fluorescence intensity over time following treatment in neurons from (**A**)). $N = 3$ / $n = 15$ neurons per condition. P-values: 10 min DMSO iLTP vs DMSO Ctrl/CHX Ctrl <0.0001; 20 min DMSO iLTP vs DMSO Ctrl/CHX Ctrl <0.0001, CHX iLTP vs DMSO Ctrl/CHX Ctrl <0.0001; 30 min DMSO iLTP vs DMSO Ctrl/CHX Ctrl <0.0001, CHX iLTP vs DMSO Ctrl/CHX Ctrl <0.0001; 45 min DMSO iLTP vs DMSO Ctrl/CHX Ctrl <0.0001, CHX iLTP vs DMSO Ctrl/CHX Ctrl <0.0001; 60 min DMSO iLTP vs DMSO Ctrl/CHX Ctrl <0.0001, CHX iLTP vs DMSO Ctrl/CHX Ctrl <0.0001; 90 min DMSO iLTP vs DMSO Ctrl/CHX Ctrl/CHX iLTP <0.0001. (**C**) Paired measurements of GPHN cluster density in dendrites prior to (−5 min) and 90 min following treatment. $N = 3$ / $n = 15$ neurons per condition. P-values ($t = -5$ min vs $t = 90$ min): GPHN density DMSO Ctrl = 0.9874, GPHN density DMSO iLTP <0.0001, GPHN density CHX Ctrl = 0.9996, GPHN density CHX iLTP >0.9999. (**D**) Paired measurements of VGAT cluster area (left) and density (right) in dendrites prior to (−5 min) and 90 min following treatment. $N = 3/n = 15$ neurons per condition. P-values ($t = -5$ min vs t = 90 min): VGAT area DMSO Ctrl = 0.6649, VGAT area DMSO iLTP <0.0001, VGAT area CHX Ctrl = 0.9999, VGAT area CHX iLTP = 0.9557; VGAT density DMSO Ctrl = 0.9893, VGAT density DMSO iLTP <0.0001, VGAT density CHX Ctrl >0.9999, VGAT density CHX iLTP = 0.9256. (**E**) Paired measurements of VGAT cluster area (left) and density (right) in miRCon or miR153 OE neurons (as seen in Fig. 5A) prior to (−5 min) and 90 min following treatment. $N = 3$ / $n = 15$ neurons per condition. P-values ($t = -5$ min vs $t = 90$ min): VGAT area miRCon Ctrl = 0.9966, VGAT area miRCon iLTP = 0.0004, VGAT area miR153 Ctrl = 0.9684, VGAT area miR153 iLTP = 0.9991; VGAT density miRCon Ctrl = 0.9975, VGAT density miRCon iLTP < 0.0001, VGAT density miR153 Ctrl = 0.5521, VGAT density miR153 iLTP > 0.9999. (**F**) Paired measurements of VGAT cluster area (left) and density (right) in treated neurons (as seen in Fig. 6D) prior to (−5 min) and 90 min following treatment. $N = 3/n = 15$ neurons per condition. P-values ($t = -5$ min vs $t = 90$ min): VGAT area DMSO Ctrl = 0.9176, VGAT area DMSO iLTP = 0.0032, VGAT area CsA Ctrl = 0.9442, VGAT area CsA iLTP = 0.9715; VGAT density DMSO Ctrl = 0.9176, VGAT density DMSO iLTP < 0.0001, VGAT density CsA Ctrl = 0.9840, VGAT density CsA iLTP = 0.9986. N = independent neuronal cultures/experiments. All values represent mean ± SEM. $*p < 0.05$, $**p < 0.01$, $***p < 0.005$, $****p < 0.0001$; mixed-effects model with Geisser-Greenhouse correction (**B**) and Šidák's multiple comparisons post-hoc test (**B–F**).

 © The Author(s)
